# Supplementary figures and images for: Plasma Exchange versus Intravenous Immunoglobulin in Worsening Myasthenia Gravis: A Systematic Review and Meta-Analysis with Special Attention to Faster Relapse Control
Source: Biomedicines. 2023 Nov 29;11(12):3180. doi: 10.3390/biomedicines11123180 (PMC10740589; doi:10.3390/biomedicines11123180)

**Suppl. Fig. 1**  
Funnel plots for change in QMGS (mean difference)

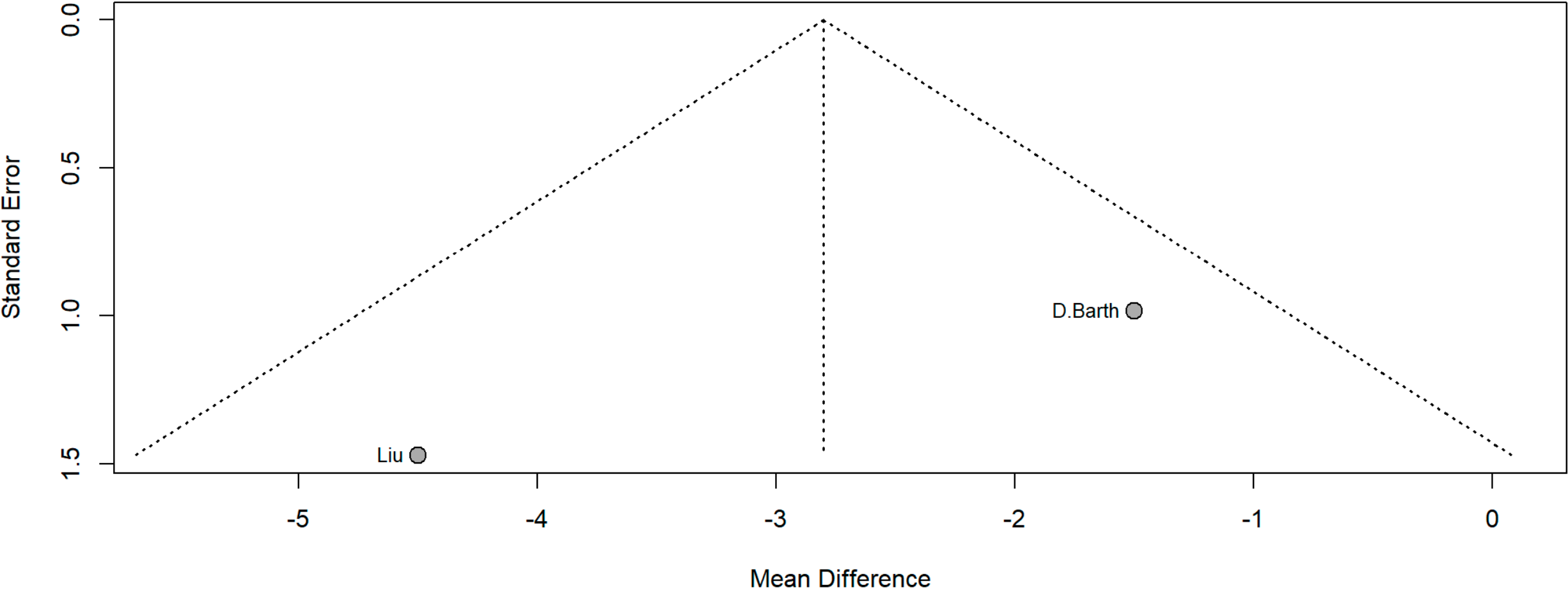

Supplement: Supplementary file 1 [file biomedicines-11-03180-s001.zip › Supplementary Figure 1.pdf]

**Suppl. Fig. 2**

Funnel plots for change in QMG (odds ratio)

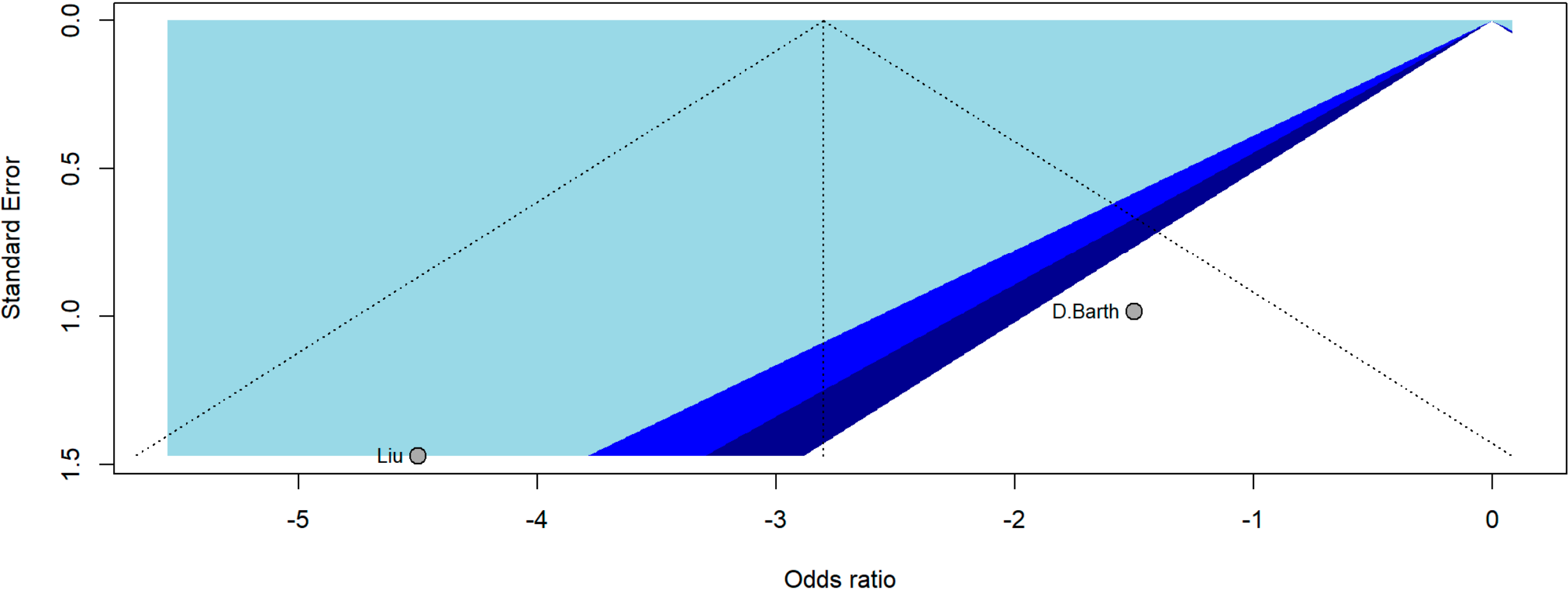

Supplement: Supplementary file 1 [file biomedicines-11-03180-s001.zip › Supplementary Figure 2 docx.pdf]

**Suppl. Fig 3**  
Funnel plots for adverse events (odds ratio V1.)

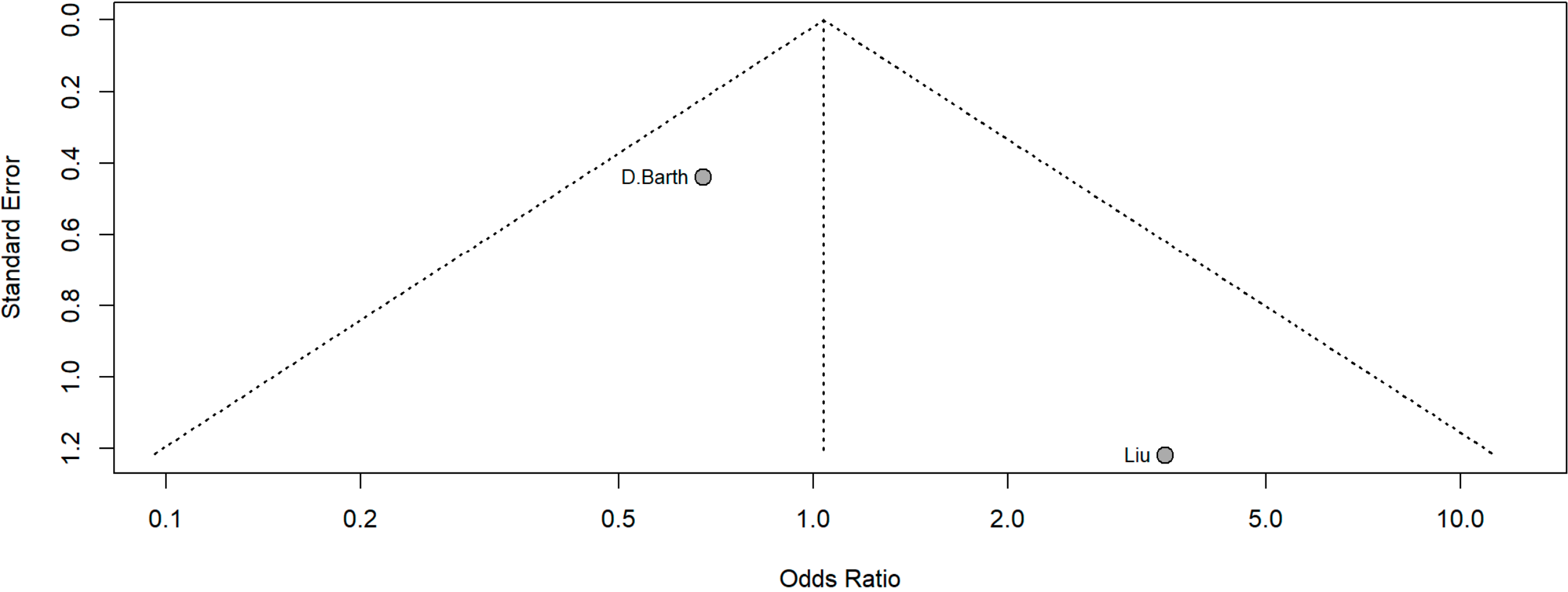

Supplement: Supplementary file 1 [file biomedicines-11-03180-s001.zip › Supplementary Figure 3.pdf]

**Suppl. Fig. 4**  
Funnel plots for adverse events (Odds ratio V2.)

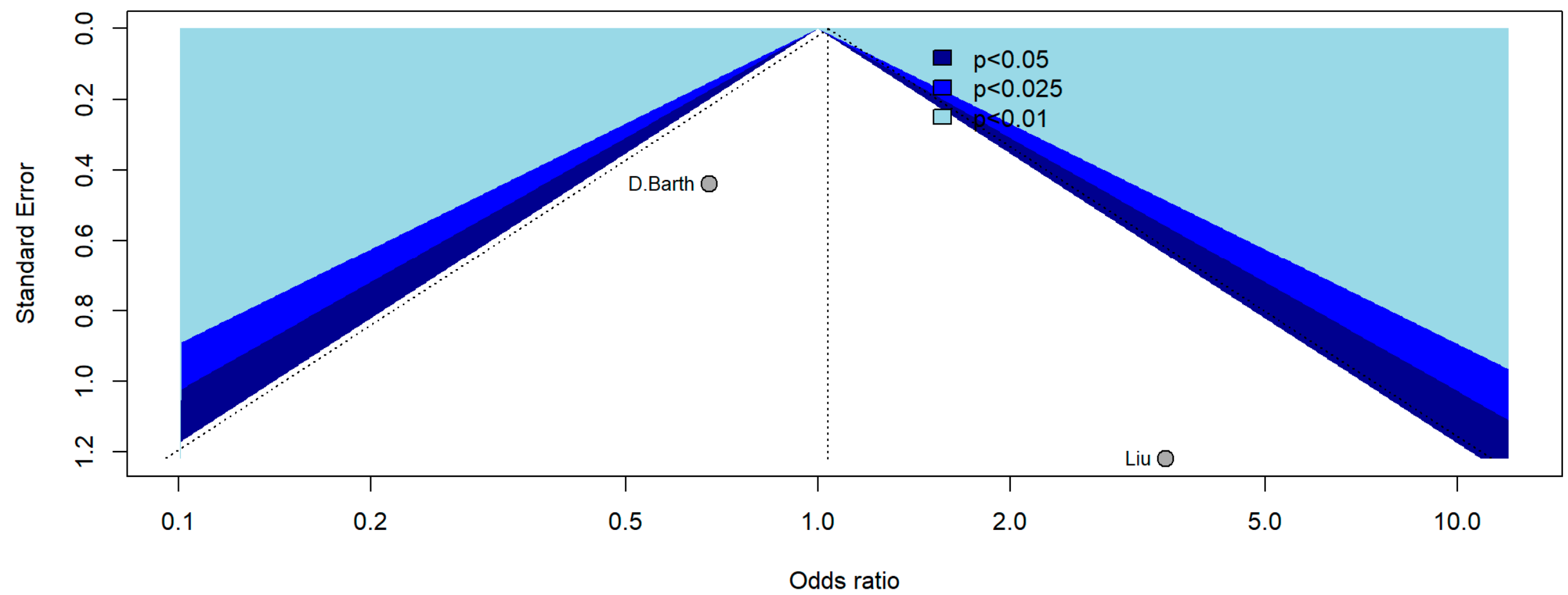

Supplement: Supplementary file 1 [file biomedicines-11-03180-s001.zip › Supplementary Figure 4.pdf]
